# Differential Diagnosis of Endometriosis by Ultrasound: A Rising Challenge

**DOI:** 10.3390/diagnostics10100848

**Published:** 2020-10-20

**Authors:** Marco Scioscia, Bruna A. Virgilio, Antonio Simone Laganà, Tommaso Bernardini, Nicola Fattizzi, Manuela Neri, Stefano Guerriero

**Affiliations:** 1 Department of Obstetrics and Gynecology, Policlinico Hospital, 35031 Abano Terme, PD, Italy; marcoscioscia@gmail.com (M.S.); bruna81@tiscali.it (B.A.V.); tbernardini@casacura.it (T.B.); nfattizzi@casacura.it (N.F.); 2 Department of Obstetrics and Gynecology, “Filippo Del Ponte” Hospital, University of Insubria, 21100 Varese, VA, Italy; 3 Obstetrics and Gynecology, University of Cagliari, 09124 Cagliari, CA, Italy; manu.neri11@hotmail.it (M.N.); gineca.sguerriero@tiscali.it (S.G.); 4 Department of Obstetrics and Gynecology, Azienda Ospedaliero Universitaria, Policlinico Universitario Duilio Casula, 09045 Monserrato, CA, Italy

**Keywords:** endometriosis, adenomyosis, bowel, rectum, ovary, bladder, ureter, abdominal wall, vagina

## Abstract

Ultrasound is an effective tool to detect and characterize endometriosis lesions. Variances in endometriosis lesions’ appearance and distorted anatomy secondary to adhesions and fibrosis present as major difficulties during the complete sonographic evaluation of pelvic endometriosis. Currently, differential diagnosis of endometriosis to distinguish it from other diseases represents the hardest challenge and affects subsequent treatment. Several gynecological and non-gynecological conditions can mimic deep-infiltrating endometriosis. For example, abdominopelvic endometriosis may present as atypical lesions by ultrasound. Here, we present an overview of benign and malignant diseases that may resemble endometriosis of the internal genitalia, bowels, bladder, ureter, peritoneum, retroperitoneum, as well as less common locations. An accurate diagnosis of endometriosis has significant clinical impact and is important for appropriate treatment.

## 1. Introduction

Endometriosis is a common, chronic, and debilitating gynecological condition that affects between 5–15% of women within their reproductive ages [1,2]. It is characterized by the growth of tissue that mimics endometrial tissue and exhibits the same responses to hormonal changes [1]. However, this abnormal tissue growth occurs outside the uterus, usually on other organs inside the pelvis and abdominal cavity, thus creating endometriosis implants. These endometriosis implants lead to local inflammatory reactions that promote fibrosis and adhesion formation, which create resistance between organs and may result in an altered pelvic anatomy [3]. This cascade of events often causes menstrual and/or chronic pelvic pain, infertility, or malfunction of the affected abdominopelvic organs [1].

In the past decades, ultrasound has become a valuable tool to accompany pelvic bimanual examinations. Currently, both methods are used for the first-line examination and diagnosis of endometriosis [4]. When a patient presents with persistent symptoms of suspected endometriosis, a comprehensive and detailed evaluation of the pelvis by ultrasound is usually prescribed. However, the accuracy of the ultrasound in detecting deep-infiltrating endometriosis depends on the experience of the sonographer. Variances in endometriosis lesion appearance and distorted anatomy secondary to adhesions and fibrosis present as major difficulties during the complete sonographic evaluation of pelvic endometriosis [5].

**Figure 1:**
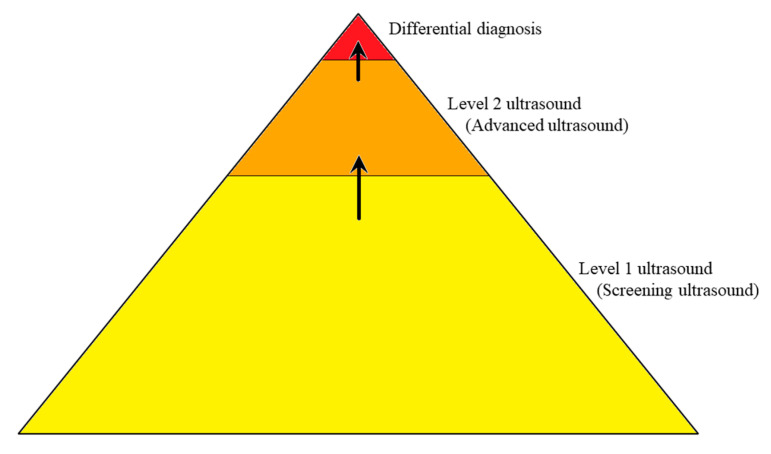
A pyramid chart that represents the distribution of ultrasound scans for endometriosis according to complexity. Although differential diagnosis must be considered in each exam, the number of cases where it makes a difference is relatively small, although it is fundamental.

Therefore, a trained ultrasound operator is needed to detect most of the endometriosis lesions, and is required to make sound judgments and to plan the appropriate treatment [5,6]. It is also important for expert evaluations to include a differential diagnosis (DD) of endometriosis to exclude all other abdominopelvic diseases. Although misdiagnoses represent a small number of cases with unknown prevalence (Figure 1), the clinical impact of a DD is great because the appropriate treatment largely varies based on the diagnosis.

## 2. What a Comprehensive Ultrasound for Endometriosis Should Investigate

Traditionally, a routine pelvic ultrasound only evaluates uterine and ovarian lesions, which leads to the detection of elementary lesions, such as endometriomas [7]. However, the evaluation should include the anterior and posterior pelvic compartments to assess the mobility of pelvic organs [7,8,9,10] and to improve the assessment of endometriosis, as suggested by the International Deep Endometriosis Analysis (IDEA) consensus [11]. The use of a systematic ultrasound approach can enhance the detection of different endometriosis locations within the pelvis [12,13,14], with a detection rate that is similar to that of magnetic resonance [15,16].

**Figure 2:**
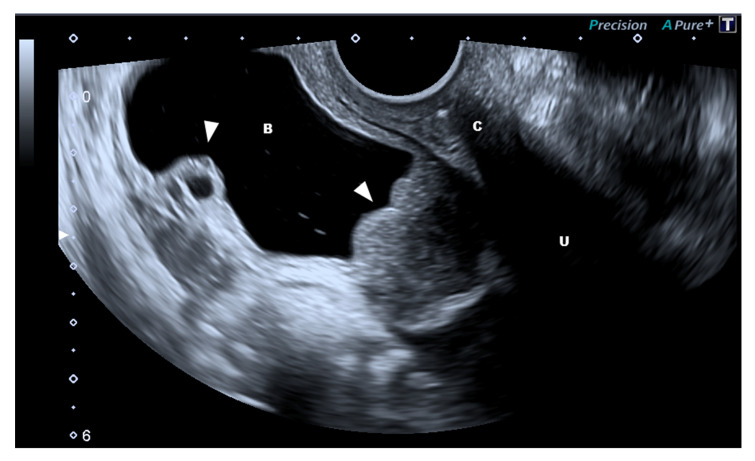
Bifocal endometriosis of the bladder (arrow). The two lesions present different characteristics as the one on the right-hand side presents a typical solid aspect while the left one is a cystic endometriosis location. Abbreviations: B, bladder; C, cervix; U, uterus.

**Figure 3:**
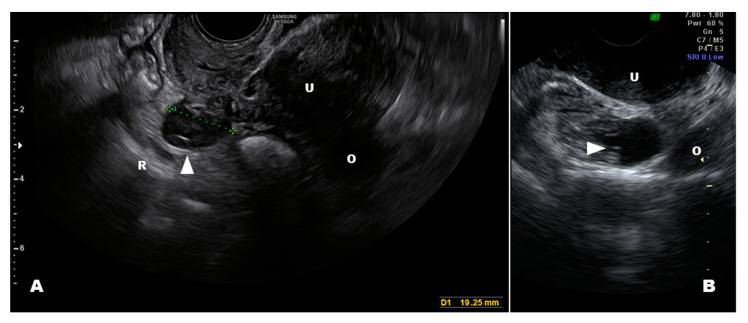
Cystic endometriosis (arrow) of the retroperitoneum in two patients on medical therapy with previous pelvic surgery for endometriosis. (**A**) shows an endometriosis cyst of the rectovaginal septum and (**B**) shows a cyst of the mesometrium. Abbreviations: U, uterus; R, rectum; O, Ovary.

Endometriosis can extend to organs other than internal genitalia, such as the bowels, bladder [1,16], and retroperitoneal structures (e.g., ureters, parametria, nerves) [17], which significantly complicates the ultrasound evaluation [18]. Although all these locations appear similar on an ultrasound (nodular, sometimes flat, hypoechoic lesions with the absence of colored Doppler spots), and they present specific features according to the organ or tissue involved. In fact, deep-infiltrating endometriosis (DIE) of hollow organs induces a retraction of margins with a subsequent irregular profile of both external and internal surfaces (i.e., bladder, bowels, and vagina), whereas DIE of dense organs (i.e., peritoneal and retroperitoneal organs) maintains a nodular hypoechoic structure [11]. Less frequently, some lesions may display vascularity with small cysts (i.e., bladder; Figure 2). Cystic endometriosis of retroperitoneal organs is a rare event that mainly occurs in patients with a history of pelvic surgery (Figure 3).

## 3. Sonographic Differential Diagnosis

During a comprehensive evaluation, the sonographer should be able to correctly identify all significant endometriosis lesions, the presence of other benign diseases (i.e., fibroids, uterine malformations, etc.), and any suspected malignancies. It is also important that the DD includes examination for adenomyosis [12,19,20,21] and endometrioma [22,23]. It is far more difficult for gynecologists to identify and characterize diseases of organs other than the internal genitalia. However, the ultrasound operator must be competent in identifying not only normal and altered pelvic anatomy due to endometriosis and other gynecological diseases, but also other diseases that are typically identified by other specialists (e.g., radiologist, gastroenterologist, urologist). Furthermore, gynecologists also have to consider that the presence of endometriosis does not exclude other clinical conditions that may require further evaluation by another specialist.

Several gynecological and non-gynecological conditions can mimic DIE, which are summarized in Table 1. In some cases, clinical signs can help differentiate diseases even when a pelvic bimanual examination is suggestive of endometriosis. Several studies have demonstrated the possibility of a DD of endometriosis lesions with benign (e.g., adenomyoma vs. myoma or endometrioma vs. other cysts) [19,22,24,25,26] and malignant genital diseases (e.g., ovarian cancer) [26,27].

### 3.1. Internal Genitalia

The ovary is the most common site of endometriosis (Table 1). Additionally, the “typical” endometrioma in premenopausal women is the easiest to identify by ultrasound due to its specific characteristics (e.g., unilocular cysts with ground-glass echogenicity of the fluid content, polar clot/debris, and poor vascularization on color Doppler evaluation) [28,29]. This should be differentiated by hemorrhagic corpora lutea especially when fine strands of fibrin are seen in the fluid content of the cyst, through the evidence of rich peripheral vascularization and its transient nature. Sometimes a correct diagnosis is not easy, especially in the presence of multiple cysts, because it can be very difficult to distinguish an endometrioma from other adnexal masses, such as some dermoid cysts, hemorrhagic cysts, tubovarian abscesses, mucinous cystadenomas, or ovarian cystic adenomyomas [24,25,26]. The presence of intracystic vascularization certainly poses doubts about malignancy (e.g., borderline tumor, endometrioid cancer) [23] also because 1% of the masses presumed to be endometriomas are malignant [30]. Furthermore, the ultrasound characteristics of pre- and postmenopausal endometriomas differ. Postmenopausal endometriomas are less frequently unilocular and are less likely to have ground-glass content; however, those with typical ground-glass content have a high risk of malignancy [28].

Superficial endometriosis of the fallopian tubes is undetectable, but it can be suspected by the presence of a tubovarian complex with tube dilatation at the location of the endometriomas. Sonographic diagnosis of tubovarian abscesses is not easy in such cases, but the clinical presentations of tube wall edema and positive color/power Doppler intensities are suggestive [31], regardless of the presence of an endometrioma (abscess superimposed to the endometriosis tubovarian complex). Solid localizations of the fallopian tubes are certainly difficult to detect but they do not require a DD in the presence of other pelvic endometriosis lesions.

Adenomyosis is characterized by an altered junctional zone with an area that is isoechoic to the endometrial tissue (for the deepening of ectopic endometrial glands and stroma), and its ultrasound classification has been well described [19,32]. It can often present as an irregularity of the junctional zone (e.g., echogenic subendometrial lines and buds, an interrupted junctional zone) with or without myometrial cystic lesions, which appear either as focal or diffuse lesions or as adenomyomas. Solid lesions, which are referred to as buds, that originate from the endometrium must be differentiated from non-endophytic endometrial cancers, such as uterine clear-cell carcinoma and uterine papillary serous carcinoma. Buds do not develop from endometrial hyperplasia, but instead they arise from an atrophic endometrium [33]. The age of onset, which largely overlaps [34], clinical presentation [35], and endometrial thickness (women with symptomatic adenomyosis or perimenopausal menstrual alterations are often on hormonal therapy) may not help in the DD; however, the myometrial vascularization may provide some clues. In adenomyosis cases, the vessel distribution (i.e., arcuate, radial, and basal arteries) within the myometrium and the lesion is not altered [19,32], whereas the neoangiogenesis that occurs in tumoral lesions is detected by color/power Doppler and the identification of aberrant vessels, even in cases of endometrial cancer in which the endometrium does not appear particularly irregular [36].

Adenomyomas are defined as benign tumors that include components derived from endometrial glands, stroma, muscular cells, and fibrosis [20]. These lesions are mostly found within the myometrium, and their sonographic characteristics resemble those of myomas. Nevertheless, in some cases they can be differentiated from myomas [21]. The correct preoperative diagnosis between myomas and adenomyomas is of key importance for surgeons because the surgical removal of adenomyomas is very difficult, if not impossible. The ultrasound characteristics of adenomyomas have been reported [12,19], and their DD mainly consists of the absence of both the pseudocapsule and vascularization compared with that of myomas. In fact, non-encapsulated myomas that lack the typical refraction at the mass border maintain the typical circumferential vascularization of fibroids, whereas adenomyomas exhibit an anatomical myometrial pattern of vessels (i.e., arcuate, radial, and basal arteries). Lacunas are not necessarily a sign of adenomyomas because uterine fibroids may exhibit cystic degeneration secondary to a limited blood supply. In fact, the thick hyperechoic wall of the cystic glands of adenomyomas is poorly represented during hormonal therapy, thus a clear DD may not be possible. Clinical symptoms may support the findings of the sonographic examination because uterine myomas with cystic degeneration are poorly symptomatic, unless they are submucosal. However, adenomyomas may present with abnormal uterine bleeding and periodic pain during ovulation and menstruations. In cases of uterine masses with strong vascularity, usually hypervascular myomas, a DD that includes adenomyosis, occult degeneration (leiomyosarcoma or smooth uterine muscle of uncertain malignant potential [STUMP]), or a benign fibroid is not possible [21].

Endometriosis of the vagina occurs in approximately 12% of women with revised American Society for Reproductive Medicine (rASRM) stage IV endometriosis [17]. It presents as a nodular thickening of the vaginal wall that causes pain under a gentle pressure with the probe [37] and that does not modify with probe compression [22]. It infiltrates the recto-vaginal septum, usually in the posterior or lateral-posterior upper third of the vagina, and it presents as a hypoechoic lesion with a negative/minimal color Doppler signaling. Its DD must include advanced cervical cancer with vaginal infiltration. The normal appearance of the uterine cervix with different vascularization as well as symptoms after intercourse (endometriosis is associated to dyspareunia, whereas cervical cancer is associated with postcoital bleeding) can lead to a correct diagnosis. A DD that includes rare tumors, such as those of primary vaginal cancer, is mainly clinical because of its clinical appearance and the age of diagnosis (more frequent in postmenopausal women).

Endometriosis of the vagina rarely presents as cystic and is usually a part of a larger solid nodulus. Vaginal cysts have been reported to occur in approximately 1% of all women, and they are located in the anterolateral vaginal wall [38]. These cysts are asymptomatic remnants of the Wolffian duct and present as regular hypo- or anechoic cysts. Less frequently, they can be located on the posterolateral vaginal wall within vaginal scars (i.e., episiotomy and birth lacerations). The key aspects for a DD are the location (endometriosis is always on the posterior vaginal wall) and the absence of tenderness.

### 3.2. Peritoneal and Retroperitoneal Endometriosis Lesions

The pelvic peritoneum is almost always involved in advanced-stage endometriosis [3,17]. The typical appearance is a nodular thickening of the peritoneum, which is sometimes nodular or flat, like a plaque (this is very often found beneath the ovary on the ovarian fossa), and hypoechoic with negative color Doppler signaling. These lesions may grow inward into tissues from a superficial site of origin, deepening further into the mesometrium (endophytic growth) and reaching subperitoneal structures, such as ligaments (e.g., uterosacral and round), ureters, and superficial nerves (e.g., hypogastric nerve). The DD includes peritoneal carcinomatosis that presents as nodular or sheet-like, exophytic, hypoechoic, vascularized structures and is almost always associated with ascites [39].

The peritoneum over the uterus and bladder can be clearly evaluated by a transvaginal scan with an unemptied bladder, and any lesions with enough thickness (nodule) can be identified [40]. Although superficial, plain lesions cannot be detected, alterations in the peritoneal profile due to retractive fibrosis and adhesions have sometimes been reported [22]. The presence of adhesions in prevesical peritoneum due to a cesarean section with the absence of the uterine sliding sign are often misleading and impossible to differentiate from endometriosis. The two clinical conditions (adhesions and endometriosis) can coexist, and it is important to evaluate if a peritoneal lesion of endometriosis deepens into the vesicovaginal septum to the bladder.

**Figure 4:**
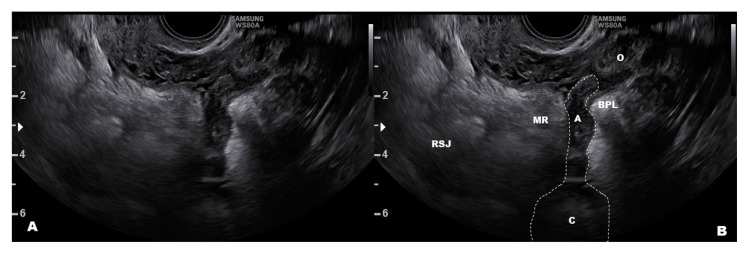
Appendix dislocated downward and attached to the ovary that may mimic mesorectal endometriosis. (**A**,**B**) show the clean and the labelled image. Abbreviations: A, appendix; O, Ovary; BPL, broad posterior ligament; C, caecum; MR, mesorectum; RSJ, rectosigmoid junction.

Retroperitoneal endometriosis near the uterus (i.e., paracervical and parametrial) is common [17,41], and ultrasound is a valid tool to identify these lesions [11,42,43,44,45]. A DD that includes the spread of cervical cancer warrants attention [46]. Endometriosis of the broad posterior ligaments may infiltrate deeply into the mesometrium and paracervical tissues, reaching organs, such as the ureter [47], and appearing as a solid nodule in most cases. Retroperitoneal cystic endometriosis is not very common (Figure 3B), and it typically occurs in women who previously had pelvic surgery for endometriosis. However, it should be differentiated from a pelvic varicocele. A misplaced appendix may be easily confused with endometriosis of the mesorectum, which is usually superficial and presents as a peritoneal implant of endometriosis (Figure 4) [48]. Cystic endometriosis beneath the rectum is very rare [49] compared with benign cysts of sacral nerve roots.

### 3.3. The Bowels

Colonic endometriosis is not a rare condition. It affects 37% of women with severe endometriosis [13], and its correct diagnosis is essential for the proper planning of treatment [15,48,50,51]. Recently, the ultrasound detection rate of endometriosis foci of the bowels has increased, exhibiting both high sensitivity and specificity [46,52]. Additionally, its accuracy is as high as other imaging techniques, such as magnetic resonance, rectal endoscopy sonography, and double contrast barium enema [11,12,15,53]. Bowel lesions can present as different shapes; however, they exhibit an anechoic appearance without posterior enhancement, they can invade the bowel lumen, and they can have digitiform, irregular, or smooth limits. The sliding sign with the uterus and near organs is often negative because the nodule may involve the Douglas pouch or the uterosacral ligaments, or it can be attached to the posterior wall of the uterus.

The DD of bowel endometriosis using ultrasound is particularly challenging for gynecologists who are not used to examining lesions other than those of endometriosis. In some cases, bowel disease may resemble endometriosis, and the DD is more difficult [54]. It is important to note that rectosigmoid endometriosis always involves the anterior or the lateral rectosigmoid wall and never the posterior wall that is directed toward the mesorectum/mesosigmoid [54]. The most common bowel diseases that can be mistaken for endometriosis include appendicitis, appendiceal tumors [48], colonic polyps, diverticulosis, and colonic malignancy [54]. Specific factors of the internal surface of the gut should be considered during a DD, such as colonic polyps and malignancies. As we reported in a previous article [54], colon cancer growths typically extend outward and reach the serosa, whereas endometriosis lesions grow inward. A transrectal ultrasound with a radial probe that scans the entire 360 degrees of the rectal lumen is a useful tool to detect and confirm the presence of polyps or malignancies; however, a colonoscopy remains the gold standard for diagnosis. In advanced-stage rectal cancer, there may be infiltration of the entire bowel wall, but the presence of specific symptoms (e.g., rectal bleeding, unexplained weight loss, gas pains or cramps) may help with the correct diagnosis.

It may also be difficult to distinguish bowel endometriosis from colonic diverticula [54]. Diverticula are mainly characterized as outward growths on a thick bowel wall with hyperechoic content, whereas endometriosis is endophytic and hypoechoic. The imaging may even be more complex if inflammation occurs within a closed diverticular abscess. In this case, an undefined oval mass that is growing outward beyond the bowel surface may be identified. Additionally, a transverse section may exhibit intense Doppler positivity in acute diverticulitis. Clinical symptoms (e.g., constant abdominal tenderness, nausea and vomiting, and pyrexia) and inflammatory blood tests may support the sonographic findings and lead to the correct diagnosis and treatment.

### 3.4. The Bladder and Ureter

Urinary tract endometriosis occurs in 0.3–12% of women with endometriosis [55,56], with an incidence of 43% in women with stage IV endometriosis [17]. Endometriosis of the muscularis propria of the bladder and the ureters are the rarest conditions, with incidences of 4.3% and 9.5%, respectively [17]. The bladder wall can be examined by ultrasound because it is surrounded by a low-echogenic tissue (i.e., perivesical loose connective tissue) and the peritoneum, which appears as a hyperechoic line. In most cases, a hypoechoic lesion that alters the profile of the peritoneum of the bladder and infiltrates into the detrusor muscle indicates bladder endometriosis and originates from uterine adenomyosis [50]. Endometriosis of the bladder is often symptomatic (e.g., urgency, frequency, and pain on passing urine, which worsen days before and during menstruation, and recurrent urinary tract infections) and appears as a consistently solid mass that involves the prevesical space, the serosa (adventitia), and the detrusor muscle, sometimes up to the urothelium [22,42,57]. These lesions always involve the bladder dome on the sagittal plane, and sometimes the nodule may also extend laterally. Additionally, they exhibit color Doppler positivity. The vascularity of these lesions may suggest a malignant origin of the nodule; however, only advanced-stage cancer (either a bladder or cervical carcinoma) invades the prevesical peritoneum. Furthermore, bladder cancer is typically found in postmenopausal women and originates from the internal surface (urothelium) of the lateral wall of the bladder. In cases of advanced-stage cervical cancer, the lesion clearly involves the cervix and quite often the lateral parametrial and paracervical tissues [50,58]. Endometriosis of the bladder may present some uncommon features, such as cystic lesions and multifocal endometriosis. In such cases, it is very difficult to differentiate from bladder cancer that may be both cystic and multifocal [59,60]. Vesical cancer usually presents as a solid papillary mass that projects into the bladder, with a normal thickness of the bladder wall at the site of the lesion [60]. Nevertheless, large carcinomas may exhibit anechoic cystic degeneration within the mass and infiltration of the detrusor muscle [60]. Color Doppler imaging is not useful for this condition because cystic endometriosis of the bladder usually shows no vascularization. When making the DD, the facts that bladder carcinomas typically occur in elderly women and that urinary cytology is the simplest, less-invasive, and least expensive method to obtain a correct diagnosis warrants consideration [50].

**Figure 5:**
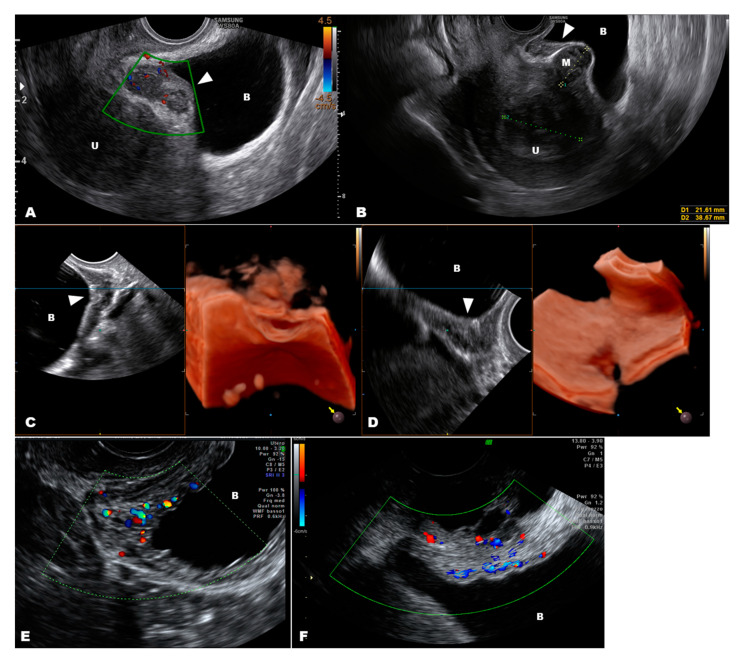
A typical endometriosis nodule (arrow) of the bladder (**A**); figure (**B**) shows a flat nodule of endometriosis (arrow) with a subserosal myoma that distorts the bladder dome; a flat endometriosis nodule (arrow) of the bladder that can be seen at 3D ultrasound only when cut (**C**) while the internal surface of the bladder appears regular (**D**); figure (**E**) shows a thick bladder wall due to cystocele and figure (**F**) in case of recurrent cystitis. Abbreviations: U, uterus; B, bladder; M, myoma.

We recently reviewed the DD of urinary tract endometriosis [50]. The DD may sometimes be difficult due to the presence of external masses that encroach the bladder dome (i.e., uterine fibroids or an attached small bowel from a previous surgery, such as a cesarean section), an intravesical ureterocele that may resemble deep lesions of endometriosis, or hypertrophic areas of the bladder wall due to a chronic, complete cystocele and recurrent cystitis [50]. In such cases, although bladder endometriosis very often appears as an endophytic lesion, it may sometimes appear flat and may be confused with hypertrophic areas due to chronic mechanical trauma (cystocele) or recurrent cystitis (Figure 5).

Endometriosis of the ureter affects the pelvic segment of the organ as a deepening of foci of the posterior broad ligament. Endometrial tissue may only invade the outer adventitia (extrinsic type) or the mucosal and/or muscular layers of the ureter (intrinsic type) [22,61]. It presents as a nodule along the course of the ureter that causes a dilatation of the ureteral tract proximal to the stenosis. The dilated ureter appears as a blood vessel in the parametrial tissue with negative color Doppler signaling and evidence of peristalsis [47], which is also often associated with hydronephrosis. The presence of ureteric calculi, which cause intense symptoms and appear as solid echogenic masses often with dilatation of the proximal part of the ureter, must be excluded [50].

### 3.5. Uncommon Endometriosis Locations

Uncommon locations of endometriosis are difficult to detect, and the accuracy of the ultrasound may be significantly reduced due to an inexperienced operator [62]. Endometriosis of the external genitalia (e.g., the perineum and mons pubis) may be easily confused with other conditions (i.e., abscesses, genital Crohn’s disease, skin trauma/swollen lesions, and hematomas) [63,64] because it may originate from abdominal wall lesions (i.e., muscular strains, heparin hematomas, muscular tumors, and hernias). In such cases, patient history in addition to the ultrasound findings is useful for identification of any conditions that resemble endometriosis [65,66,67]. The rarest sites of endometriosis include lesions of the liver, pancreas, kidney, and gallbladder. Hepatic endometriosis appears as superficial hypoechoic lesions and may mimic an abscess, a hematoma, an angioma, or malignancy [62]. Endometriosis of pancreas, kidney, and gallbladder show hypoechoic lesions that can be seen by ultrasound and they require a second evaluation by computerized tomography or magnetic resonance imaging [62]. These lesions do not present pathognomonic features that may differentiate endometriosis from malignancy or adenomas, so the definitive diagnosis relies on histology only [62].

## 4. Conclusions

The accuracy of ultrasound for the diagnosis of pelvic endometriosis has greatly improved over recent years, and it is widely regarded as the first-line diagnostic tool, despite a significant interobserver variability has been reported [68]. Certainly, the identification of extra-genitalia lesions requires a specific training [69]; however, ongoing practice is necessary to acquire and maintain competence [70].

DD by ultrasound for patients who have been referred for endometriosis may result in the detection of other gynecological diseases; therefore, it is important to consider other conditions when unusual lesions are detected. A skilled operator is key because his mental reconstruction of the distorted anatomy is necessary to correctly localize and evaluate the lesion of interest. Additional knowledge of the ultrasound appearances of lesions that do not occur within the reproductive system is needed to make a correct diagnosis. however, in such cases, further examinations should be required, or patients should be referred to other experts/specialists. A specific hands-on training for making DDs is not possible, but theoretical courses and off-line scanning sessions for sonographers with experience in gynecological and endometriosis ultrasound help to improve their background and may result in more accurate diagnoses.

## Figures and Tables

**Table 1:** Endometriosis possible anatomic locations, anatomical features, and differential diagnosis.

Anatomic Locations	Anatomical Aspect	Possible Differential Diagnosis
**Internal genitalia**		
Ovary	Cystic	Hemorrhagic corpus luteus Hemorrhagic cyst Dermoid cyst Tubovarian abscess Mucinous cystoadenoma Ovarian cystic adenomyoma Malignancy
Tube	Cystic	Tubovarian abscess
Uterus (adenomyosis)	Focal, solid (buds)	Endometrial malignancy
	Solid adenomyoma	Non-encapsulated myomas
	Cystic adenomyoma	Myomas with cystic degeneration/necrosis Suspected sarcoma/STUMP
Vagina	Solid nodulus Cystic	Cervical malignancy Vaginal malignancy Benign vaginal cyst
**Peritoneum**		
Broad anterior/posterior ligament	Solid	Peritoneal carcinomatosis
Douglas pouch	Solid	Peritoneal carcinomatosis Advanced cervical malignancy
Prevesical peritoneum	Solid	Post-cesarean adhesions Peritoneal carcinomatosis Advanced cervical malignancy
Uterosacral ligaments	Solid	Peritoneal carcinomatosis Advanced cervical malignancy
Round ligaments	Solid	Peritoneal carcinomatosis Advanced endometrial malignancy
**Retroperitoneal tissues**		
Paracervix/parametrium	Solid	Advanced cervical malignancy
Mesometrium	Solid Cystic	Pedunculated infraligamentary fibroid Ovarian malignancy Varicocele
Mesorectum	Solid Cystic	Colonic malignancy Downward dislocated appendix Retrorectal dermoid cyst
Presacral space	Cystic	Tarlov cyst Ganglioneuroma
Vesicovaginal septum	Solid	Bladder malignancy
**Bowel**		
Rectosigmoid	Solid	Colonic polyp Diverticulosis Colonic malignancy
Caecum and appendix	Solid	Appendicitis Appendiceal tumors
Ileum	Solid	Malignancy
**Urinary tract**		
Bladder	Solid Cystic	Bladder malignancy Cervical malignancy Uterine leiomyomas/adenomyomas Hypertrophic traumatic bladder Bladder malignancy Intravesical ureterocele Small bowel attached to the prevesical peritoneum
Ureter	Solid	Ureteric stone
**External genitalia**		
Perineum	Solid	Postpatum scar Chron disease
Mons pubis	Solid	Abscess Hematoma Muscular strain
**Abdominal wall**		
Umbilicus	Solid	Omphalitis Umbilical granuloma
Abdominal muscle	Solid Cystic	Strain Benign tumor (desmoid) Hematoma
Inguinal canal	Solid	Complete small hernia
